# Strengthening the Use of International Collaborative Regulatory Assessments and Regulatory Alignment– Implications for Global Convergence

**DOI:** 10.1007/s43441-025-00817-8

**Published:** 2025-06-08

**Authors:** John H. Skerritt, Mark Mayer, Jeffrey Francer

**Affiliations:** 1 Faculty of Medicine, Dentistry and Health Sciences, University of Melbourne, Melbourne, Victoria 3010 Australia; 2 Eli Lilly and Company, Lilly Corporate Center, Indianapolis, IN 46285 USA

**Keywords:** Regulation, Evaluation, Collaboration, Reliance, Recognition, Facilitated pathway

## Abstract

There has been a significant growth in international regulatory information-sharing and work-sharing initiatives in recent years, leading to reductions in submission lag and regulatory review timeframes. However, regulatory approvals in some major countries can still lag by months or years after the first global approval, with impacts on availability of new medicines for patients. This review assesses the impact of current international collaborative initiatives and proposes some options for their advancement. It also explores the potential impact of other factors such as greater alignment and collaboration on facilitated pathways, Good Manufacturing Practice) GMP inspections on regulatory timeframes and makes suggestions for improvements of regulatory convergence, collaboration, reliance and administrative procedures. While international collaborative regulatory assessments are still relatively new, in the following years, we consider that these pathways will become even more routine and impactful, especially if they can be further adapted.

## Introduction

Patients in many countries can often wait some years until a medicine with regulatory approval from the United States Food and Drug Administration (US FDA) is available locally. While especially a problem in low- and middle-income countries, significant delays to market access are also a common challenge in high-income countries. Delays arise from both regulatory and patient access issues (through government reimbursement or insurance programs), but the first step in reducing delays is to reduce regulatory approval times. The Centre for Innovation in Regulatory Science (CIRS) has published metrics on the timing of regulatory approval and first health technology assessment decisions in markets such as Australia, Canada, Singapore, Switzerland and the United Kingdom [1–3].

In recent years, several international collaborative initiatives have had a positive impact on regulatory review timeframes, without compromising the evaluation of product safety, quality and efficacy. These include the implementation of facilitated review pathways, international collaborative review and regulatory reliance and recognition processes. Despite these innovations, for most medicines there remains a significant lag in regulatory approval between the US, European Union and Japan and other countries or regions. In this paper, we review the performance of these initiatives and make some suggestions for their potential evolution. Our focus is on new medicine/ new active substance submissions and label extensions in markets with well-established regulatory agencies, namely the regulatory agencies to which international companies typically make the first set of regulatory submissions. The relevant agencies include US FDA, EMA (European Medicines Agency) / EU regulatory system, PMDA (Pharmaceutical and Medical Devices Agency) Japan, Health Canada, MHRA (Medicines and Healthcare products Regulatory Agency) UK, Swissmedic, TGA (Therapeutic Goods Administration) Australia, NMPA (National Medical Products Administration) China, MFDS (Ministry of Food and Drug Safety) Korea and ANVISA (Agência Nacional de Vigilância Sanitária) Brazil. This is variable, however, as larger pharma companies may include additional countries in their initial regulatory submission planning while small biotech companies may focus only on the US and European markets.

We initially look at some established and emerging international regulatory collaborations, assess the potential for recognition and reliance to be used more widely and finally explore some other approaches that may reduce time to regulatory approval. The challenges that lack of alignment between the facilitated regulatory pathways in different countries creates is also discussed.

### Established International Regulatory Consortia

Several initiatives have been formalized in recent years to provide structured collaboration between different regulatory agencies. Several such as US FDA’s Project Orbis and the European Medicines Agency (EMA) OPEN initiative focus on information sharing while the Access Consortium undertakes work sharing. The emphasis of this paper is on a subset of major markets (namely US, EU, Canada, UK, Japan, Australia, Switzerland, Korea, Brazil and Singapore) although there are also active collaborative initiatives in the Caribbean, ASEAN (Association of South East Asian Nations) and Africa. “Information sharing” involves close communication between regulators during the review of a product and/or after its regulatory approval. “Work sharing” involves different regulators simultaneously evaluating different parts of the dossier, with intermediate exchange of information and discussions during the separate evaluations to produce a combined evaluation report.

### The Access Consortium

Access is a coalition of medium-sized regulatory authorities that work together to promote greater regulatory collaboration and alignment of regulatory requirements [4]. Members include the Therapeutic Goods Administration (TGA) of Australia, Health Canada, Health Sciences Authority (HSA) of Singapore, Swissmedic and the UK’s Medicines and Healthcare products Regulatory Agency (MHRA, since October 2020). Access partners undertake information and work-sharing across several product and technical areas, although new active substance (NAS) drug licensure, particularly including extensions of indications (but in some cases also line extensions) is most relevant here.

In a workshared review, each regulator independently evaluates the administrative and country specific parts of the dossier (Modules 1 and 2). Modules 3 to 5 are typically only reviewed by a single agency with assessment reports and lists of questions developed. The collaborating regulators undertake a peer review to develop a consolidated list of questions. Following completion of the review each agency makes an individual (sovereign) approval or rejection decision. As of the end of 2023, 60 NAS have been approved through Access work-sharing [5], although there is not a single public database of approved products. Australia and Canada have participated in the largest number of collaborative evaluations to date.

### Impact of Access

The CIRS [2, 5] have assessed the impact of Access work-sharing on the timing of regulatory decisions made in the period 2019–2023. The major impact was a very significant decrease in the median submission lag (i.e. from the date of submission to the first regulator (usually FDA) and submission to the Access regulator). Submission lags were reduced by a median of 374 days (TGA); 272 days (Health Canada); 257 days (Swissmedic) and 347 days (HSA) compared with non-Access drugs. Therefore, the submissions were received 68–93 days after the FDA for Access medicines compared with 330–467 days for all other drugs. The review time for work-shared evaluations under Access was reduced but often not by much– in Canada 5 days and Australia 27 days but by 86 days in Singapore and 102 days in Switzerland. Therefore, the impact of Access in reducing evaluation timeframes differed across participating regulators.

Patients, companies and regulatory agencies have all benefited from Access [6]. Apart from faster regulatory approvals, potentially leading to earlier patient access, there is a saving of regulatory agency resources. Regulators and industry sponsors usually only need to manage a single set of questions on the submission, and while regulators make sovereign decisions, there is a greater chance of decision alignment. Several Access partners publish public assessment reports to enable transparency of regulatory decisions. Access reviews also cover a wide range of medicines (drugs and biologics from several therapeutic areas).

### Potential Enhancements to Access

A 2022 industry survey [5] recommended that the Access processes could be enhanced by ensuring review timelines are predictable, improving guidance documents, simplifying processes for submission and regulator interaction (including the need to make sure that a single rather than rolling set of questions is provided), increasing transparency of the review process, and establishing a process for formal industry engagement on policies and processes.

Encouragingly, Access has recently expanded pathway applicability to include medicines accepted for priority review, but we recommend that drugs designated for conditional/ provisional approval also be included. While Access information-sharing collaboration on vaccines and drugs for COVID-19 was invaluable, the work-sharing approach was determined to be too slow to be used during the pandemic. It may be possible to reduce Access timeframes by reducing the rather lengthy notification periods currently required ahead of the submission date, implementing common electronic platforms for regulatory collaboration (these are currently under development by Health Canada) and streamlining post-review procedures in some Access countries, such as Switzerland. The submission dossier (except for the country-specific module) is required to be identical across all partners. Some Access partners require very extensive Chemistry, Manufacturing and Controls (CMC) data which can mean that the evaluation is more focused on these issues and that subsequent post approval changes can be extensive. This CMC divergence can result in companies not pursuing an Access submission entirely or to only engage with some of the participating Access countries for a particular medicine. Apart from the need to change internal mindsets, some of the resistance to using Access more extensively by companies relates to their regulatory teams being organized along geographical regional lines which can make it hard for the company to work with the Access consortium which operates across four continents.

The Access consortium membership has not expanded to include additional regulatory authorities since the MHRA joined in 2020. It may not be feasible to significantly expand full Access membership at this time, including because several regulatory agencies work in languages other than English or may have limited capacity to workshare. We suggest that official “Observer” status could be considered, as is available in other regulatory consortia, to expand the reach of Access activities. For example, Brazil, Mexico, New Zealand, China, Taiwan (Chinese Taipei) and South Korea may have an interest in involvement in Access provided that suitable confidentiality agreements were executed. If Access members consider value in incorporating “Observer” status, it may be advantageous to initiate this through a pilot approach to gain experiential insights.

### Project Orbis

Orbis is an initiative of the US FDA Oncology Center of Excellence (OCE) that aims to give patients faster access to promising cancer treatments across several countries [7]. Project Orbis commenced in May 2019 and now involves US, Canada, Australia, Brazil, Israel, Singapore, Switzerland and the UK as full members. Project Orbis regulator partners work together on the review of cancer medicines with reviews benefiting from a wider range of technical inputs. The emphasis of Project Orbis is on NASs and new indications for oncology drugs where there is evidence of ability to address unmet medical needs or offering significant improvement over existing therapies. Countries make sovereign decisions about each approval. Only a third of Orbis approvals have been for new molecular entities, the majority being for line extensions.

**Fig. 1:**
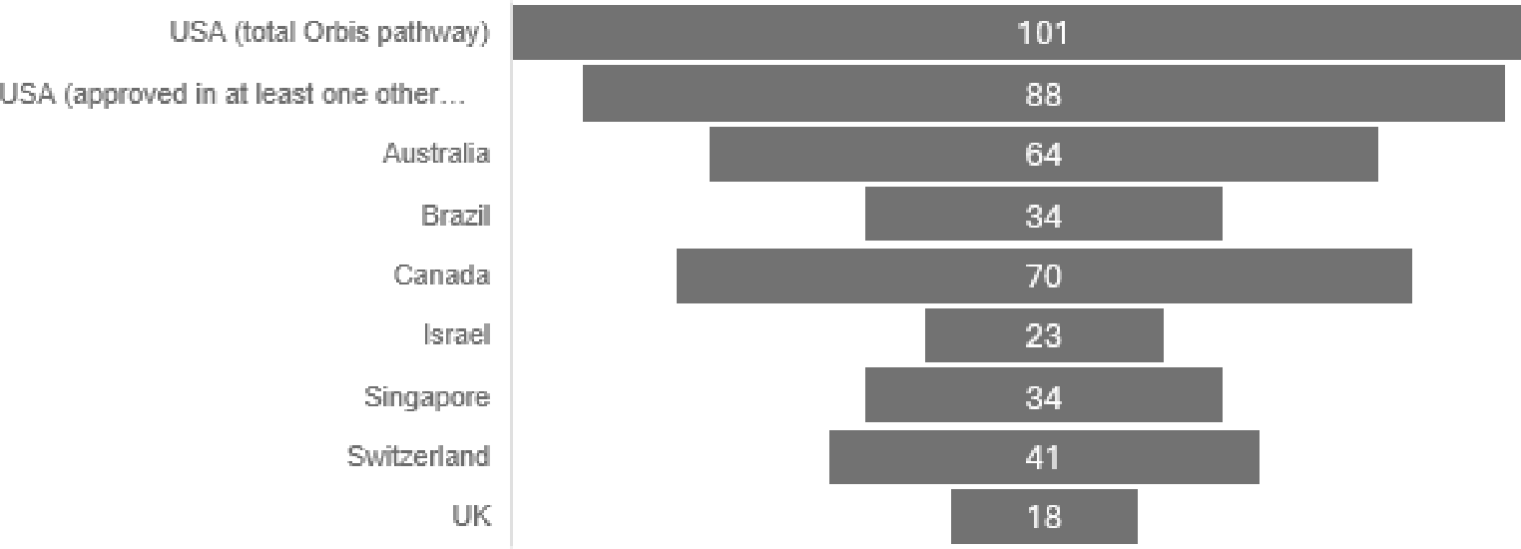
Project Orbis approvals in each participating country^1 1^ as of August 2024, data calculated from reference [7]

The US FDA manages a public database on project Orbis approvals. Although limited to oncology medicines, it is noteworthy that many more drugs have been through Orbis pathways than Access. As of early September 2024, the US FDA has approved 101 oncology medicines through the Orbis pathway [8], with 88 having an approval in one or more other countries (the remainder are still under evaluation in one or more countries). Orbis approvals have been greatest by the two original partners, Australia (64) and Canada (70), but there have been significant numbers in other partners (Fig. 1).

There are three types of Project Orbis submissions:

- Type A - where submission to the partner is largely concurrent.
- Type B - where there is more than a 30 day delay from FDA to partner submission.
- Type C - where submission to the partner typically occurs after FDA approval.

Under Orbis A and B the partner country participates in multi-country review meetings and attends FDA review meetings, but not under type C. In effect Pathway C (which is the most commonly-used pathway overall) [2, 3] is an information-sharing pathway rather than a collaborative review pathway.

### Impact of Project Orbis

Submission lags were decreased relative to non-Orbis oncology medicines although the reduction was typically not as great as for Access reviews [2, 3]. Submission lag relative to FDA was reduced by 395 days (TGA); 165 days (Health Canada); 233 days (Swissmedic), 39 days (HSA), 90 days (ANVISA) and 75 days (Israel). On the other hand, review times for Orbis oncology medicines compared with other oncology medicines were usually (but not always) reduced to a greater extent than under the Access program, by 77 days (Health Canada); 120 days (Swissmedic) and 94 days (HSA). However median review times were 2, 23 and 50 days longer for project Orbis drugs for Israel, TGA and ANVISA respectively. FDA’s own approval times were faster for project Orbis drugs (215 days versus 240 days for non-Orbis medicines) as they had priority review despite the additional work required by the FDA to coordinate Project Orbis. Access by medium-sized agencies to the extensive oncology resources within FDA can increase confidence and support earlier decision making. FDA’s own considerations are also enhanced through international collaboration. The discussion of uncertainties between agencies may resolve issues faster than relying upon sponsor questions. Other studies have also shown that involvement in Project Orbis can increase concordance in regulatory decisions between agencies.

It would be valuable to assess other potential impacts of Orbis (and Access). For example, whether for medicines reviewed through these programs there is greater alignment of medicine indications and safety warnings between participating countries. Also, are there corresponding efficiencies achieved in regulatory review or follow on indications and/or efficiencies gained for subsequent health technology assessments where applicable?

### Potential Enhancements to Project Orbis

The requirement that the medicine must qualify for FDA priority review to be included in Project Orbis could be reconsidered, as there are several new cancer drugs without priority designation that may have significant therapeutic benefits. To enable earlier regulatory decisions, more Orbis A and B (rather than Orbis C applications) should be encouraged to enable simultaneous review. Expansion of Orbis country participation or observer status could be considered, e.g. South Korea, Taiwan and Mexico. Most impactful would be the wider implementation of similar FDA-led international collaborative schemes for biologics and (non-oncology) medicines. Orbis and potentially other schemes should also be open to all New Active Substance and line extensions.

### Other International Collaboration Programs

The EMA OPEN initiative involves participant evaluation of medicines in parallel with EMA but with significant information-sharing and participation in certain EMA evaluation meetings, including the CHMP (Committee for Medicinal Products for Human Use) [10]. It started solely collaborating on COVID-19 therapeutics and vaccines, with a pilot including Australia, Canada, Japan, Switzerland and the World Health Organization (WHO). In 2023, scope was expanded to include drugs for antimicrobial resistance, drugs designated under the EMA “PRIME” priority medicines scheme or with a designated high unmet medical need and those addressing public health threats and emergencies. South Korea and Brazil have also recently joined. Benefits for collaborating countries appear similar to participation in Orbis, but there are no statistics yet on whether OPEN has led to more rapid regulatory approvals by collaborators, although it is expected that participation reduces submission lag. Involvement in the OPEN initiative has been welcomed by the participating countries and medicine developers. It may be possible to expand participation to countries such as Israel and Mexico. While recognising that the additional administrative burden on the EMA, consideration could be given to extending OPEN to all NAS types.

A separate initiative, the CoGenT (Collaboration on Gene Therapies Global Pilot) [11] focussing on gene therapies has been recently piloted as an additional international collaborative mechanism. Led by FDA and involving EMA, Japan, Health Canada and Swissmedic, it has some similarities to Project Orbis. Partners may participate in FDA regulatory meetings and meetings with the sponsor, and FDA’s regulatory reviews are shared and discussed with partners. It aims to reduce the timeframes and increase efficiency of the regulatory process.

### Streamlining Regulatory Reliance and Recognition

Recognition has been defined as [12] “Acceptance of the regulatory decision of another regulator…based on evidence that the regulatory requirements of the reference regulatory authority are sufficient to meet the regulatory requirements of the relying authority”. Among medium-large regulators (others can be taken to include US FDA, EMA, Health Canada, TGA Australia, Swissmedic, HSA Singapore, PMDA Japan, NMPA China, MFDS Korea, ANVISA Brazil), it is only utilized by Mexico and more recently, the UK [13] and New Zealand [14]. Singapore has a verification route [15] with a 110 working day total timeframe (screening plus evaluation) which has elements of both a reliance and a recognition pathway. The MHRA “Recognition A” review timeframe of 60 days would potentially enable approval very soon after US FDA or EMA approval.

Reliance has been defined [12] as “the act whereby the regulatory authority in one jurisdiction takes into account and gives significant weight to assessments performed by another regulatory authority… in reaching its own decision”. Evaluations from a reference country (usually EMA and US FDA; often also PMDA, Health Canada, TGA and Switzerland) are used to speed regulatory review. While reliance processes were predominantly formerly utilized in low and middle-income countries (LMICs), several established regulatory authorities (including in UK, Switzerland, Australia, NZ, Mexico, Brazil, Israel, Colombia, Taiwan and Singapore) have adopted them, while Canada is commencing a reliance pathway for pediatric drugs [16]. While reliance necessarily means the reference regulator must have first completed their review, it could still be the fastest path to market for less-resourced regulators such as Singapore, New Zealand, Colombia and Mexico.

### Potential Enhancements to Reliance and Regulatory Recognition

Although there may be resistance to the introduction of recognition pathways in other countries, the UK “Recognition A” pathway could be an exemplar if several current exclusions were limited. For example, products are excluded if they have first-in-class new active substances or cell and tissue therapies, are orphan drugs or have conditional regulatory approval elsewhere. They are also excluded where an environmental risk assessment has not been conducted by the reference agency or where the clinical trials utilized single-arm designs and/or real-world evidence. The proposed New Zealand recognition pathway also will require prior approvals from two, rather than one major regulator, which will reduce its effectiveness in reducing approval times.

Reliance has been successfully employed in many countries, particularly when prior approval is required from one, rather than two countries. Challenges relating to the two countries that typically are first to approve medicines internationally (namely obtaining unredacted reports from FDA and limited access to translated reports from Japan) mean that EMA is the main reliance partner. Current reforms to reduce overall approval times in the EU should increase attractiveness of reliance [19]. Where the same company is the sponsor for applications in other countries, approaches could be improved to enable more ready access to unredacted FDA summary basis of approval documentation, such as only requiring redaction when the commercially confidential information (CCI) is owned by another party rather than the medicine’s sponsor.

Some reliance process timelines could be reduced, particularly for pathways where the submission dossier and all aspects of the product are identical to the reference country and comprehensive evaluation reports are submitted. There is also the need for a single database, possibly held by the WHO or the International Coalition of Medicines Regulatory Authorities (ICMRA) and replicated on individual regulators’ websites, to map out regulatory reliance arrangements by country. The US-based FRPath project provides reasonably extensive information on facilitated regulatory (including reliance) pathways [20].

### Alignment of Facilitated Regulatory Pathways

Many well-established regulatory authorities have implemented facilitated pathways (FRP) [18–21]. CIRS analysis [2] showed that from 2018 to 2022, the usage of facilitated regulatory pathways (priority review, provisional/ conditional approvals, regulatory reliance, worksharing and information sharing) had increased for most of the agencies compared with 2013–2017. FDA was the agency that used facilitated pathways to the greatest extent, with 75% of NASs being reviewed through at least one FRP, followed by Health Canada (51%), Swissmedic (51%), TGA (48%), PMDA (38%) and EMA (36%).

While having some overall similarities in requirements e.g. for priority review or provisional/conditional approvals, the specific criteria, processes and benefits of the pathways to applicants are not aligned between countries [19]. There also seem to be international differences in how “unmet medical need”, one of the main criteria for access to an FRP is assessed, as use of facilitated pathways differ significantly between countries. In 2022, the ratio of expedited approvals to standard reviews was highest for FDA (71%), followed by PMDA (39%), Health Canada (22%), EMA (10%), Swissmedic (8%), and TGA (6%) [2]. EMA had the greatest differences in median approval time between expedited and standard review in 2022, with 183 days, whereas the smallest difference was for PMDA, 57 days. The difference between standard and expedited review was 132 days for Health Canada, 117 days for TGA, 113 days for Swissmedic, and 94 days for FDA.

Provisional or conditional approval of medicines enables earlier access to market for highly promising products, in many cases based on the results of phase 2 rather than phase 3 trials. This is a common feature e.g. of FDA accelerated approval, EMA conditional approval, PMDA conditional early approval and TGA provisional approval, together with the need for the medicine to address unmet clinical need and for confirmatory trials to be undertaken. However, there are differences between the criteria applied and processes used. Some of these include differences in the seriousness of condition for the medicine to qualify (e.g. FDA and Health Canada– serious condition versus EMA and TGA– severe or life-threatening condition); whether the preliminary data must show an advance over alternate medicines (TGA) and the timing of the decision for the medicine to be considered for provisional approval (before (FDA, HC, TGA) versus during (EMA) review).

The lack of consistent criteria can therefore add to the complexity of product development and regulatory management if it means that only some markets enable much earlier access through conditional or provisional approval. Facilitated pathways also seem to be applied differently to different medicine classes and therapeutic areas although this is not usually specified in guidelines or regulation. For example, the proportion of oncology drugs that were granted facilitated pathway review significantly exceeded neurology products [22].

### Potential Enhancements to Facilitated Review

While recognising that regulatory changes may be required, we believe that earlier regulatory approvals across global markets could be achieved if there was greater consistency in requirements and practices between markets for access into expedited pathways, which are today similar but not identical in many countries. Eligibility criteria for a product for facilitated pathways must also be clear and consistently applied. Demonstrating eligibility is sometimes more straightforward for first-in-class products. It is sometimes hard to demonstrate significantly greater efficacy or safety in the absence of direct comparisons between products, a feature which is not usually present in regulatory submissions. There is potential to more systematically explore the potential for greater use of priority submissions, particularly with EMA and other regulators outside US FDA and PMDA. This may require the ability to engage in dialog ahead of a formal pre-submission meeting, although most regulators will be keen to see convincing results from phase 3 trials before making any form of preliminary commitment to priority review. The participation of countries in different international programs, and/or existence of facilitated or reliance review pathways is summarized in Table 1.

**Table 1:** Summary of the regulatory pathways available in selected countries

Jurisdiction	Pathways available					
	Access	Project Orbis	EMA OPEN (full member)	Priority Review	Provisional/ conditional/ FDA accelerated pathways	Reliance or recognition
USA		**X**		**X**	**X**	
Canada	**X**	**X**	**X**	**X**	**X**	
Brazil		**X**	**X**	**X**		**X**
Mexico						**X**
Colombia						**X**
Europe (EMA)		**#**	**X**	**X**	**X**	
UK	**X**	**X**		**X**	**X**	**X**
Switzerland	**X**	**X**	**X**	**X**		**X**
Israel		**X**		**X**		**X**
Japan			**X**	**X**	**X**	
South Korea			**X**	**X**		
Taiwan (Chinese Taipei)				**X**		**X**
China				**X**	**X**	
Singapore	**X**	**X**				**X**
Australia	**X**	**X**	**X**	**X**	**X**	**X**
sNew Zealand				**X**		**X**

1. Priority review here is defined as more rapid full review of a drug by the named regulator (i.e. not utilizing regulatory reliance)

2. May only apply to certain classes of therapeutic products in some countries (e.g. drugs but not biologicals in Australia). Some additional countries have provisional or conditional pathways applicable to products in public health emergencies

3. Reliance in this case only describes the situation for premarket drug or biologic review. A pediatric reliance pathway is coming soon in Canada

### Increasing the Efficiency of International Collaborative GMP Models

Delays to obtaining manufacturing approvals can lead to delays in market authorization. The Pharmaceutical Inspection Cooperation Scheme (PIC/S) is well known for leading international development, implementation and maintenance of harmonized GMP standards and quality systems for inspecting drug manufacture. PIC/S membership provides a degree of assurance over the quality of inspections but it is not a mutual recognition scheme. Several initiatives have streamlined manufacturing approvals and reduced the need for individual onsite inspections by each jurisdiction. These include:

- Mutual GMP inspection recognition agreements, such as those between US FDA and -MHRA, Swissmedic and the EMA or the “Agreement of Cooperation” for GMP inspections between US FDA and Health Canada [23].
- The collaborative hybrid inspection pilot between Access countries [4].
- Alternatives to the onsite inspection of a manufacturing site by use of recent inspection by a trusted comparable regulator through “GMP clearance”, as used widely by Canada, Australia and Singapore, for example [20, 21].

Similar initiatives should be investigated for extension to more countries, with multi-regulator inspections encouraged, including where one agency is physically present and collaborating agencies take part virtually. In addition, adoption of a single GMP license that could cover multiple sites (as implemented by Canada and the EU and encouraged by WHO) would also streamline processes.

### Other Potential Enhancements to International Regulatory Collaboration

There are several other ways in which regulators in certain countries could speed regulatory approval processes without affecting evaluation quality (Table 2).

First, administrative processes within agencies could be improved, in particular by streamlining timeframes of their pre-submission processes. Pre-submission processes are important in that they confirm that the dossier is complete before acceptance for review, these could be conducted more rapidly. The timeframes for the pre-submission processes are not typically counted in determining overall regulatory review timeframes, so these are actually longer than published. More efficient dossier screening processes could be implemented with regulatory agencies publishing clear criteria for dossier completeness requirements.

Second, while external advisory committees provide a valuable perspective, if there was greater flexibility in their timing this could save delays in regulatory approvals. Committees are often consulted late in the regulatory review process and they meet infrequently. If the committees typically met earlier in the review cycle the timing of regulatory decisions would be less dependent on the meetings calendar. Further, if the (national) committees meet late in the review cycle and raise new issues this can lead to disparity between decisions, such as those on drug labels between different countries.

Third, in the case of EU, Japan and Switzerland, improvement of processes following the end of scientific assessment could further speed final decisions. For the EU and Japan, the delay is due to final decisions being made by separate parties (the European Commission and the Japanese Ministry of Health, Labour and Welfare respectively) rather than by the regulator. In Switzerland there are delays after the preliminary decision is made due to negotiation time with the sponsor particularly around the drug label; these could instead be undertaken during the regulatory review phase.

Fourth, some regulatory agencies such as FDA, Health Canada, TGA, New Zealand, Singapore and Japan issue market authorizations indefinitely. Products stay approved, unless if the sponsor fails to pay annual charges or GMP certificates cease to be valid. In some jurisdictions, there is also a requirement for the drug to be made available on the market within a set period from market authorization. In others e.g. EMA (this will soon change), UK, Switzerland, China, South Korea, Taiwan, Brazil, Mexico and Colombia there is a requirement to renew market authorizations typically after 5 years. We contend that because companies already have whole-of- lifecycle obligations such as periodic safety update reports (PSURs) and regulators have tools to remove drugs that are unsafe or ineffective from the market, the requirement for renewals of market authorization could be reconsidered. This process can potentially decrease regulatory alignment between countries. In addition, the resources saved could be dedicated instead to streamline other market authorization and regulatory activities.

Fifth, the Certificate of Pharmaceutical Product (CPP) was established by WHO in 1969 to stimulate the development of regulatory reliance through providing documentary assurance that a particular product has been fully evaluated by the issuing regulatory authority [26]. While there have been recent enhancements, such as the development of electronic CPP templates and processes, it may be timely to review the place of CPPs in the regulatory scheme. The major reference agencies announce product approvals on their internet sites and several have registers of all approved products, duplicating some information on the CPP. The CPP requirement not only involves an additional administrative process with some direct delay but also means that in many countries the regulatory evaluation process cannot commence until the product is approved elsewhere. It also requires administrative resource to implement for regulators and medicine developers.

Finally, stronger international collaboration in some other regulatory areas could also foster earlier submission of a greater number of aligned regulatory submissions. These may include greater use of scientific advice by regulators outside FDA, EMA and PMDA and collaborative sharing of this advice. For companies planning to launch products globally, scientific advice from regulators other than FDA, EMA, PMDA (and possibly the other North Asian agencies) is often valuable. We consider that if a wider number of regulators were able to provide scientific advice (separate to, and well prior to regulatory pre-submission meetings) there would be positive impacts on review efficiency and timeliness. Regulators may have specific requirements in non-clinical areas and toxicology, and early scientific advice opportunities would provide the opportunity for greater mutual understanding of regulatory issues raised by new technologies, planned use of real world evidence or use of non-standard clinical trial designs.

A particular area for greater future regulatory alignment is in evaluation of drugs or vaccines manufactured using platform technology. A ‘drug platform’ refers to the similarities across multiple products with respect to molecular structure and product composition, non-clinical attributes, manufacturing process and product quality attributes [27] and can build upon the use of prior regulatory knowledge in manufacturing and quality assurance (QA) assays can be used to streamline development and regulatory submissions for both small and large molecules [28]. The EMA and FDA have recently consulted on development of platform technology guidance, which if implemented more broadly could streamline product development and regulatory review across a wider range of countries. However, it is likely that the two agencies will develop quite contrasting approaches [27, 29], with FDA proposing to designate products as utilizing a platform technology while EMA is developing platform CMC (chemistry manufacturing and controls) guidance and may potentially enable the use of a platform masterfile approach. We contend that it is important that opportunities for international alignment are sought for newer regulatory concepts such as platform technologies.

### Reduction of Submission Lag

The major factor contributing to later regulatory approvals in many countries is not the time taken by the regulator to conduct the review, but rather the lag between submission to the first regulator to approve a particular medicine (usually the FDA) and submission to the regulator in question. The approval times for six major regulators ranged from 322 to 430 calendar days in 2022 [2] but with an EMA submission lag of 66 days, PMDA with 71 days, Health Canada with 205 days, TGA with 229 days and Swissmedic with 270 days. Longer submission lags were observed in 2023 [3]. The regulatory review timeframes for major Asian markets were similar to those in the six regulatory authorities above (median time across 2019–2021): South Korea − 376 days, Taiwan − 393 days, Singapore − 408 days but China was slower at 513 days [29]. Median submission lags for 2019–2021 were South Korea (158 days), Taiwan (220 days) and Singapore (224 days). Median submission lags for large multinational companies with significant regulatory, manufacturing and distribution capacities are likely somewhat shorter than those for smaller companies [31].

At one level, the timing of a regulatory decision submission could be seen to be under the control of the sponsor, but there are many factors which constrain submission timing and may be out of the control of the sponsor. Several of these have been discussed above and include whether a medicine is accepted into the Access or Orbis collaborative review programs by the collaborating regulators or a national facilitated review program and whether there are country specific requirements (ranging from administrative procedures through to a formal requirement for local clinical trials). We contend that as global regulatory convergence and collaborative regulatory pathways continue to expand, there will be a corresponding reduction of the submission gap. If regulatory submissions are to be made to as many global markets as possible, simultaneously or very soon after those made to FDA, it will be critical to address not only the regulatory policy and process issues discussed in this review (summarized in Table 2) but also to modify internal company processes to support these development activities. This may include having the capacity to simultaneously handle reviewers’ questions from multiple regulators and to have supply chain and marketing infrastructure in place to enable simultaneous launch in several markets.

**Table 2:** Summary: potential enhancements to international regulatory Cooperation

Collaborative Regulatory Science Approach	Potential Regulatory Policy Enhancements
Access Consortium	Predictable review timeframes Simplify processes for submission and interaction with agencies Formal industry engagement on policies and processes Common electronic platforms for interaction with all Access regulators Introduce observer status for certain non-member countries
Project Orbis	Remove requirement that medicine must quality for FDA priority review Implement similar programs for non-oncology medicines Expansion of country participant or observer status
EMA OPEN initiative	Extend to all new active substance types Expansion of country participant or observer status
Reliance and recognition pathways	Require approval in only one, not two reference countries Reduce carve-outs for recognition pathways Enable more ready access to un-redacted FDA evaluation reports
Facilitated regulatory pathways	Align criteria and processes for pathways to a greater extent Increase alignment of the level of use of facilitated pathways between agencies Ensure pathways are applied similarly to different medicine classes and therapeutic areas
International collaborative Good Manufacturing (GMP) compliance models	Extend mutual GMP inspection recognition arrangements between trusted partners Implement collaborative hybrid inspections more widely Utilize paper-based GMP clearance approaches more widely
Technological advances	Implement cloud-based international collaboration on review of initial regulatory submissions (as well as post-approval changes) Implement collaboration efficiencies that derive from the application of artificial intelligence
Other potential enhancements	Align advisory committee processes and timing Reduce administrative processes following the completion of scientific assessment Reconsider need for renewals of market authorization Review the place of Certificates of Pharmaceutical Product (CPP) Greater collaborative sharing of scientific advice International alignment on platform technology approaches Reduction of submission lag

## Conclusions

The various formal regulatory collaboration programs which have been implemented over the last decade have already demonstrated significant impacts on the timeliness of regulatory review in participating countries, particularly when impacts on submission lag are considered. In addition, informal and formal information sharing between major regulators is at unprecedented levels [32]. Surveys of industry and regulatory agencies have for example emphasized the value of regulatory cooperation, including reliance and the importance of sharing confidential evaluation documents [33].

While it is recognised that many countries also require positive health technology and payer decisions, aiming for regulatory approval as expeditiously as feasible should still result in earlier patient access. Regulatory approval is always a necessary pre-requisite for funding of a drug through government payers or insurers. Therefore, faster regulatory approval usually leads to faster funding and thus faster patient access. Reforms to regulatory processes should be considered alongside other system improvements. These include, but are not limited to, improvements to clinical trial design and protocols including decentralized trials, that can enhance enrolment and reduce delays in trial completion [34, 35] and reforms to health technology policies and processes, noting that there is currently very significant disparity in the health technology assessment decision outcomes and timeliness between countries [36]. Establishment of, and involvement in collaborative regulatory review mechanisms typically requires an initial increase in effort by regulators and companies alike. After the first few collaborative evaluations there are usually benefits in reduction of effort. Greater process alignment may also increase the level of alignment in regulatory decisions [37–39].

Apart from expanding the involvement in international regulatory consortia to more countries (including as observers) a variety of other approaches should be considered, as outlined in this paper. To implement changes that expedite more rapid and internationally aligned regulatory evaluations it is also to understand the enabling factors that would be required for change. Some of these factors can be objectively assessed (such as whether a legislative or regulatory change is required to introduce an improved approach). Cultural factors within an agency are often hard to assess externally but they can have a major influence on whether changes will be made. Issues such as the willingness of evaluators to collaborate with another agency’s evaluators, or to accept their evaluations in a reliance or recognition paradigm, or of the senior leadership to modify regulatory administrative processes are critical.

The focus of this review has been on the initial regulatory submission and decision. There have been other successful pilots, such as that conducted by ICMRA on the assessment of CMC post-approval changes. The pilot Pharmaceutical Quality Knowledge Management System (PQKMS) capability aims to ensure timely and complete information and assessments about the state of pharmaceutical quality management and risk management capabilities. The program envisions harmonized structured regulatory information, using unique facility identifiers to support manufacturer oversight and secure sharing of information about manufacturing facilities. A longer-term aim is to move towards full harmonization of data elements in the quality modules of the common technical document (CTD) to not only streamline post approval changes but assist sponsors to make simultaneous market authorization submissions [40].

Technological advances also provide tremendous potential for enhancing global regulatory collaboration, but medicines regulators have been slower to implement digital transformation than in some other areas of regulation. Cloud-based systems, providing real-time access to a single dossier and its regulatory evaluation [40] has shown promise for assessment of post-approval CMC changes e.g. the ACCUMULUS Synergy initiative pilot [42]. These can facilitate collaboration between the sponsor and several regulators at once, but the bigger challenge will be to extend this to multi-country collaboration on review of full regulatory submissions. A second burgeoning area is use of artificial intelligence (AI) to automate and simplify regulatory processes, such as preparation of clinical study reports and dossier modules and supporting responses to evaluator questions [43]– [44]. To date, implementation of AI has been greater by companies, although regulatory agencies are assessing how AI could be best used in company-agency interactions, and what controls may be appropriate [45]. These characteristics should also enable AI applications to facilitate international regulatory collaborations.

Over the next decade, we consider that global regulatory convergence and collaborative assessment models will continue to expand due to the many benefits discussed in this paper, importantly to facilitate earlier global patient access to new medicines.

## Data Availability

No datasets were generated or analysed during the current study.
